# The Use of Mononucleosome Immunoprecipitation for Analysis of Combinatorial Histone Post-translational Modifications and Purification of Nucleosome-Interacting Proteins

**DOI:** 10.3389/fcell.2020.00331

**Published:** 2020-05-08

**Authors:** Kashif Aziz Khan, Marlee K. Ng, Peter Cheung

**Affiliations:** Department of Biology, York University, Toronto, ON, Canada

**Keywords:** nucleosome, immunoprecipitation, mononucleosome IP, MNase, combinatorial histone modifications, chromatin-binding proteins, histone variant

## Abstract

The nucleosome is the principal structural unit of chromatin. Although many studies focus on individual histone post-translational modifications (PTMs) in isolation, it is important to recognize that multiple histone PTMs can function together or cross-regulate one another within the nucleosome context. In addition, different modifications or histone-binding surfaces can synergize to stabilize the binding of nuclear factors to nucleosomes. To facilitate these types of studies, we present here a step-by-step protocol for isolating high yields of mononucleosomes for biochemical analyses. Furthermore, we discuss differences and variations of the basic protocol used in different publications and characterize the relative abundance of selected histone PTMs and chromatin-binding proteins in the different chromatin fractions obtained by this method.

## Introduction

The nucleosome is the fundamental repeating unit of chromatin in eukaryotic cells and is the main physiological state by which the functional genome engages the nuclear environment. A nucleosome typically consists of 147 bp of DNA wrapped around a histone octamer comprising two copies each of the core histones H2A, H2B, H3, and H4 (Luger et al., 1997). The composition and characteristics of nucleosomes can vary within the genome through the incorporation of histone variants or post-translational modifications (PTMs) of core and variant histones (Talbert and Henikoff, 2017). Moreover, the positioning and organization of nucleosomes over different parts of the genome can be further modulated by chromatin-remodeling complexes and chromatin-binding proteins (Lai and Pugh, 2017).

Core and linker histones are the main protein components of nucleosomes. They are highly and specifically expressed during S phase to cope with the demands of DNA replication-coupled chromatin assembly. Core histones are also ubiquitously distributed across the genome to form the general scaffold of genomic chromatin. Unlike core histones, histone variants are expressed and deposited into chromatin in a replication-independent manner (Henikoff and Smith, 2015). The distribution of histone variants can also be more targeted and localized such as the restriction of CENPA to centromeres. To date, variants of all 4 core histone types have been identified. The variant family of H2A is the most diverse and includes several members such as H2A.Z-1, H2A.Z-2, H2A.X, macroH2A1, macroH2A2, and H2A.Bbd. Other well-studied variants include H3.3, CENP-A/cenH3, H3.X, H3.Y of the H3 family, and H2BE, TSH2B, H2BFWT of the H2B family (Law and Cheung, 2013; Maze et al., 2014). More recently, an H4 variant, H4G, has also been identified in human cells (Long et al., 2019). Similar to core histones, histone variants are post-translationally modified at amino acids conserved between the variant and its core histone counterpart, or at variant-specific sequences.

The regulation and functions of histone PTMs have been heavily studied in the past 25 years (Zhao and Garcia, 2015; Stillman, 2018). Histones are modified by a variety of modifying enzymes and the modified histones, in turn, can elicit or facilitate specific downstream events. In the natural context, many combinations of histone PTMs co-exist on the same histone molecule and also on different histones within the same nucleosome. Some histone modifications are functionally coupled, such as the requirement of H2B mono-ubiquitylation for H3K4 methylation in yeast and human cells, or the coupling of H3 phosphorylation and acetylation during activation of immediate-early genes (Lee et al., 2010; Ng and Cheung, 2016). These examples illustrate that some histone PTMs cross-talk and cross-regulate one another as part of their regulatory mechanisms (Strahl and Allis, 2000; Lee et al., 2010). In addition, there are also combinations of histone PTMs that co-exist to mark distinct chromatin states. For example, although H3K4- and H3K27-methylation are respectively linked to gene activation and repression, these two histone modifications can also exist together to mark “bivalent” domains that correspond to poised but transcriptionally silenced genes in the stem cell genome. More interestingly, biochemical characterization of bivalent nucleosomes showed that the respective H3 methylation marks are located on the different H3 molecules within the same nucleosome. Therefore, nucleosomes can be heterotypically modified on the histone dimer pairs, leading to more complex combinatorial patterns of histone PTMs within chromatin (Voigt et al., 2012; Sen et al., 2016; Shema et al., 2016).

One of the functions mediated by histone PTMs is the recruitment of effector proteins via PTM-dependent interactions. The discovery of bromodomain-containing proteins binding to acetylated histones, and some chromodomain-containing proteins binding to methylated histones, led to the concept that there are families of “reader” proteins that bind specific histone modifications and are recruited to target sites in a PTM-dependent manner to execute downstream functions (Taverna et al., 2007). There is also accumulating evidence that recruitment of effector proteins can occur across multiple core and variant histones by binding to multiple epitopes within the nucleosomes. This type of “multivalent” interaction is not easily deciphered using individual histones or histone peptides alone since they could involve interactions with physically distal modifications or epitopes found on different histones. Indeed, one study that directly compared the PTM-reader interactions between peptide versus nucleosome contexts found only limited overlap of the co-purified reader proteins using the respective peptide/nucleosome baits (Nikolov et al., 2011). Similarly, many studies used affinity purification to identify histone-binding proteins and they generally do not distinguish between soluble versus nucleosomal histones. For example, in an effort to identify H2A.Z interacting proteins, many studies used whole cell extracts as a source of histones and predominantly identified chaperones and remodeling complexes that bind free H2A.Z (reviewed in Ng and Cheung, 2016). However, additional unique proteins were identified when H2A.Z in the nucleosome context was used as a bait to pull down H2A.Z interacting proteins (Fujimoto et al., 2012). Therefore, there is a need, as well as a growing interest, in studying histone-nuclear factor interactions at the nucleosomal level.

One of the approaches used to study histones in the nucleosome context is the reconstitution of nucleosomes *in vitro*. The nucleosome core particle or a nucleosome array can be reconstituted under low salt conditions using recombinant histones and recombinant DNA containing multiple repeats of a nucleosome positioning sequence such as the “601 sequence” (Lowary and Widom, 1998; Dyer et al., 2004). Alternatively, *in vitro* nucleosome assembly can also be done using ATP-dependent assembly factors such as recombinant ACF and RSF1 (Lusser and Kadonaga, 2004). In addition, chemically modified or peptide-ligated recombinant histones carrying specific PTMs have been generated that are in turn assembled into “designer” nucleosomes (Muller and Muir, 2015; Nadal et al., 2018). These approaches allow better control over the composition of the nucleosomes and produce a homogenous sample that is suitable for *in vitro* biochemical assays. However, such nucleosomes lack the complex range of PTMs normally seen in endogenous nucleosomes and may not fully replicate physiological chromatin.

**FIGURE 1:**
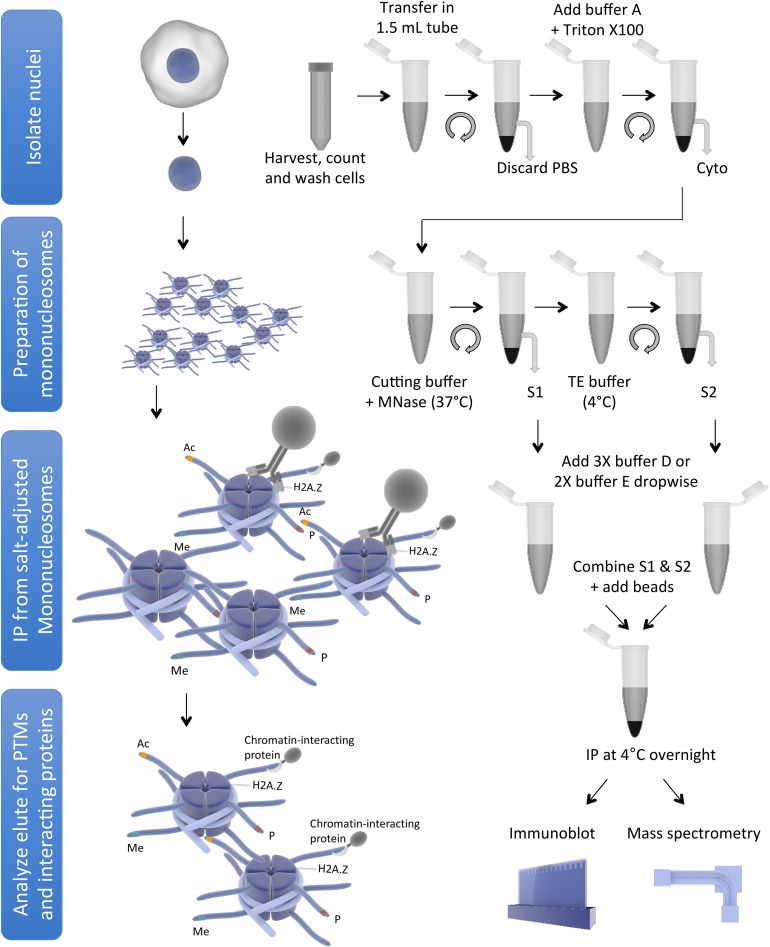
Schematic representation of mononucleosome IP protocol (for simplicity, some washing steps are not shown). The figure was created using the Library of Science and Medical Illustrations from somersault18:24 licensed under a CC BY-NC-SA 4.0 license.

Endogenous nucleosomes are historically obtained by treatment of chromatin with micrococcal nuclease (MNase), which preferentially cuts the linker DNA to generate single nucleosomes (reviewed in Kornberg, 1977), followed by immunoprecipitation (IP) of core/variant histones or histones modified by specific PTMs. Mononucleosome IP has been used by us and others to demonstrate preferential combinations of histone PTMs or histone variants that co-exist within individual nucleosomes (Sarcinella et al., 2007; Ku et al., 2012; Voigt et al., 2012; Chen et al., 2014; Lacoste et al., 2014; Wang et al., 2014, 2018; Won et al., 2015; Surface et al., 2016), or to identify proteins interacting with histone PTMs or histone variants in the nucleosome context (Draker et al., 2012; Kim et al., 2013; Sansoni et al., 2014; Vardabasso et al., 2015; Li et al., 2016; Punzeler et al., 2017; Zhang et al., 2017; Zink et al., 2017; Sun et al., 2018). In addition, the same method has been used to show incorporation of specific core/variant histone in the chromatin (Kanda et al., 1998; Wiedemann et al., 2010; Lau et al., 2011; Ruiz and Gamble, 2018), and to demonstrate effects of oncohistones on chromatin (Bender et al., 2013; Chan et al., 2013; Lewis et al., 2013; Herz et al., 2014; Fang et al., 2016; Lu et al., 2016; Piunti et al., 2017). However, there are subtle to considerable differences among the protocols used in different studies, which may lead to variations in findings, such as some differences in the H2A.Z nucleosome-interacting proteins found in different studies. We, therefore, review here the differences and variations among the protocols used by different publications to generate and immunoprecipitate mononucleosomes in order to provide direct comparisons for the readers. In addition, we also describe a mononucleosome purification and IP protocol used in our lab as a starting point for readers to test and optimize. This protocol describes a step-by-step procedure to obtain a high yield of mononucleosomes using MNase followed by IP of histone variant containing mononucleosomes. This protocol can be used to identify co-existing PTMs on histone variants and partnered core histones within the nucleosome, as well as nucleosome-interacting proteins. The schematic representation of mononucleosome IP protocol is shown in Figure 1.

## Variations and Optimization of the Mononucleosome IP Protocol

**FIGURE 2:**
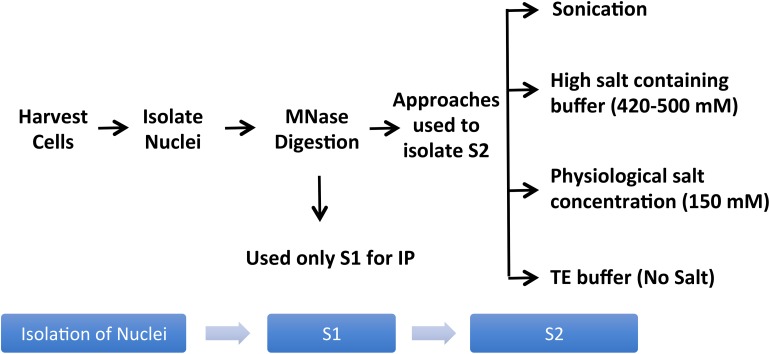
Variations of the mononucleosome IP protocol used in different publications. Pure nuclei are digested with MNase to cut the linker region followed by centrifugation to recover the MNase-digested supernatant (S1). Several studies used S1 only for IP, leaving out the insoluble material altogether (Foltz et al., 2006; Wiedemann et al., 2010; Kim et al., 2013; Chen et al., 2014; Vardabasso et al., 2015; Won et al., 2015; Li et al., 2016; Punzeler et al., 2017; Zink et al., 2017; Kim et al., 2018; Ruiz and Gamble, 2018; Sun et al., 2018). Additional steps used in the literature to obtain an S2 fraction for the maximum recovery of mononucleosomes from MNase-digested chromatin include sonication (Viens et al., 2006; Fang et al., 2015), extraction using high salt containing buffer (Kanda et al., 1998; Sarcinella et al., 2007; Lau et al., 2011; Ku et al., 2012; Voigt et al., 2012; Surface et al., 2016; Zhang et al., 2017), buffer with physiological salt concentration (Sansoni et al., 2014), or no-salt TE buffer (Draker et al., 2012).

Studies of histones at the nucleosomal level require a good yield of mononucleosomes that is typically obtained by *in nucleo* digestion of nuclei by MNase. Nuclei are isolated by swelling of cells in a hypotonic solution followed by the addition of a detergent to disrupt the cellular membrane (Mendez and Stillman, 2000). Pure nuclei are recovered by centrifugation and then digested with MNase in a CaCl_2_-containing buffer to cut the linker region, followed by centrifugation to recover the mononucleosome containing supernatant (S1). There are generally only minor differences amongst protocols used by different studies in terms of the composition of hypotonic solution or CaCl_2_-containing buffer for the digestion of nuclei by MNase to extract S1; however, there are significant differences in the approaches used to recover remaining mononucleosomes from the pellet as the 2^nd^ supernatant (S2) (Figure 2).

**FIGURE 3:**
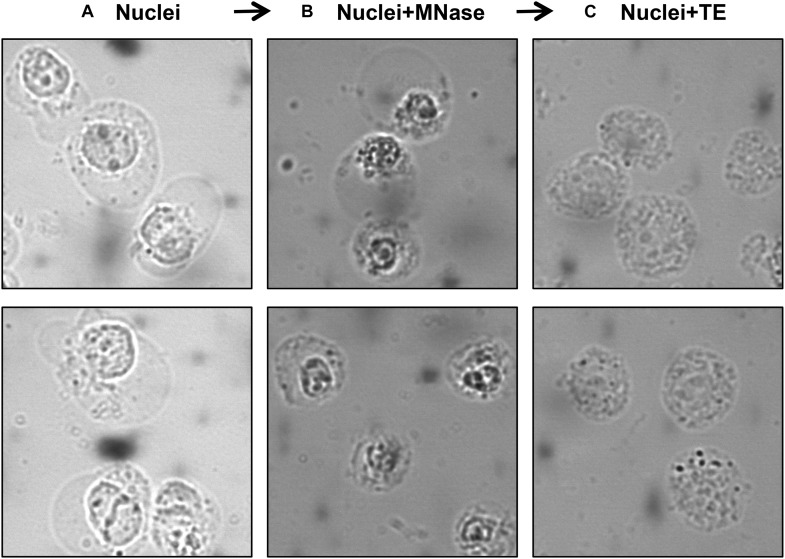
Visualization of nuclei at different steps of the protocol using a phase-contrast microscope. Isolated nuclei at Step 6 **(A)** MNase-digested nuclei at Step 9 **(B)** TE-resuspended nuclei at Step 11 **(C)**.

A number of studies used MNase-digested supernatant (S1) only for IP, leaving out the remaining chromatin and insoluble material after MNase digestion (Foltz et al., 2006; Wiedemann et al., 2010; Kim et al., 2013; Chen et al., 2014; Vardabasso et al., 2015; Won et al., 2015; Li et al., 2016; Punzeler et al., 2017; Zink et al., 2017; Kim et al., 2018; Ruiz and Gamble, 2018; Sun et al., 2018). Other studies included additional steps to obtain an S2 fraction for the maximum recovery of mononucleosomes from MNase-digested chromatin. A variety of different approaches have been used for this step; one approach is to sonicate the insoluble material to increase the yield of mononucleosomes (Viens et al., 2006; Fang et al., 2015) while another is to use a high salt-containing buffer based on the classical Dignam nuclear extraction protocol (Dignam et al., 1983) to extract residual chromatin and proteins. The latter method involves incubating the MNase-digested nuclei in 420–500 mM NaCl-containing buffer followed by centrifugation to collect S2 (Kanda et al., 1998; Sarcinella et al., 2007; Lau et al., 2011; Ku et al., 2012; Voigt et al., 2012; Surface et al., 2016; Zhang et al., 2017). Although nucleosome integrity is maintained under high salt concentration, these conditions can disrupt the interaction of chromatin-binding proteins. Moreover, we have observed significant precipitation of insoluble chromatin during the salt extraction, likely due to the mobilization of linker histone H1 under these conditions (Clark and Thomas, 1986; Al-Natour and Hassan, 2007). Therefore, for our studies of combinatorial histone PTMs and nucleosome-interacting proteins, we prefer to isolate mononucleosomes from MNase-digested nuclei under low or no salt conditions. We, like other studies, collect the mononucleosome containing supernatant S1 by centrifugation after digestion with MNase, but we then resuspend the MNase-digested nuclei pellet in salt-free TE buffer to lyse the nuclei for maximum recovery of mononucleosomes (Draker et al., 2012). As demonstrated in Figure 3, suspending intact nuclei in the no salt buffer leads to rupture of the nuclear membrane and further liberation of free intact nucleosomes.

**FIGURE 4:**
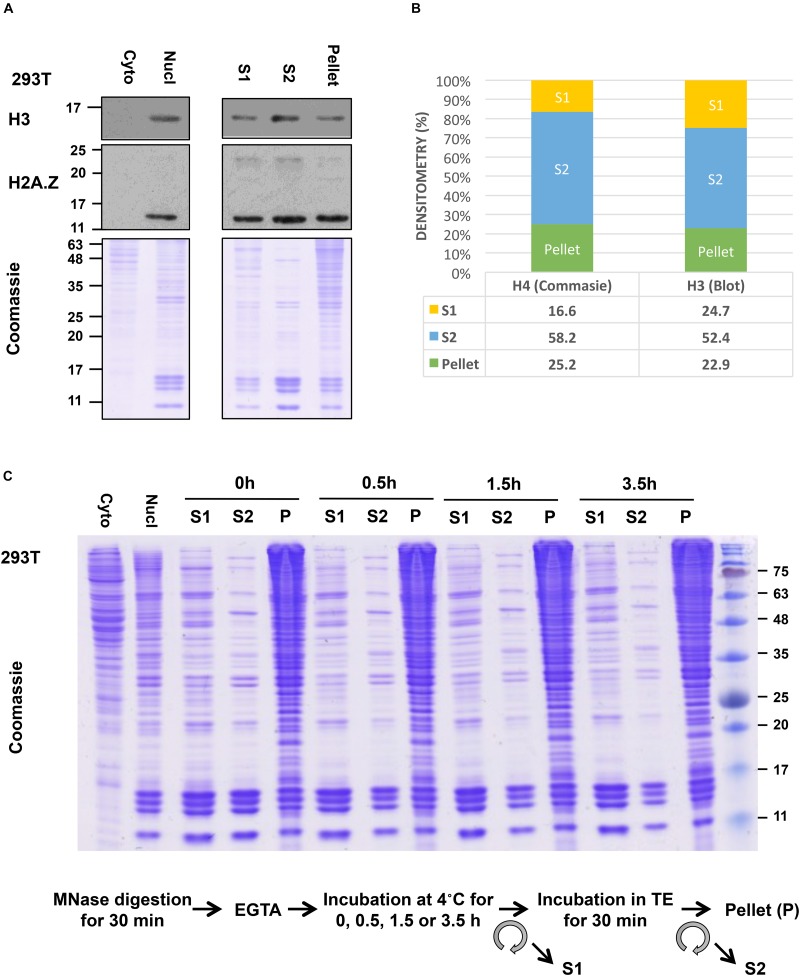
Yield of mononucleosomes in the MNase-digested (S1), the TE-soluble (S2), and the insoluble-pellet fractions. **(A)** Nuclei from 293T cells were treated with 1.0 U/10^6^ cells and different fractions from an equivalent number of cells were separated with 10–15% SDS-PAGE and blotted with indicated antibodies or stained with Coomassie stain. Cytoplasmic and nuclear extract from an equivalent number of cells was included in parallel. **(B)** Densitometry analysis of H4 band from Coomassie gel and H3 blot using ImageJ. **(C)** The nuclei were incubated for an extended period after stopping the MNase reaction with EGTA (step 9) followed by centrifugation to recover S1 and then incubated in TE buffer to isolate S2 as described in the protocol.

Although our S1 showed a good yield of mononucleosomes, our S2 fraction showed even higher yield of mononucleosomes, as evident in the side-by-side comparison of the amount of core histone proteins in these fractions by Coomassie gel staining or by the levels of histone H3 and H2A.Z in immunoblot analysis (Figure 4A). Densitometry analysis of the H4 band from Coomassie gel and H3 blot using ImageJ (Schneider et al., 2012) showed that around 75% of total histones in all 3 fractions were recovered in the combined S1 and S2 fractions (Figure 4B). Blotting of FLAG-tagged histones showed similar results (data not shown). Although some histones are still left in the insoluble pellet, this method allows us to pool both S1 and S2 fractions before IP to ensure that results are obtained using the majority of released nucleosomes. Slightly different from our method, other studies isolate mononucleosomes after a second longer incubation of the post-S1 extracted nuclei in physiological salt concentration. For example, Imhof and colleagues incubated the MNase-digested nuclei in 150 mM NaCl containing buffer with end-to-end rotation overnight and centrifuged to recover S2. Nonetheless, in both cases, the S1 and S2 fractions were pooled before IP (Sansoni et al., 2014).

**FIGURE 5:**
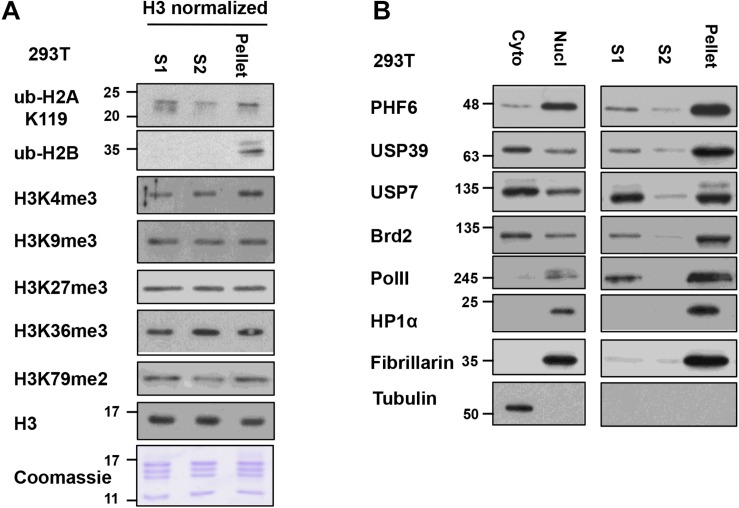
Relative levels of histone post-translational modifications (PTMs) and nucleosome-interacting proteins in S1, S2, and insoluble-pellet: **(A)** The quantity of nucleosomes in the S1, S2, and the insoluble pellet were normalized based on the H3 signal and Coomassie-stained gel and then analyzed for the levels of the various histone modifications by immunoblot analysis. **(B)** S1, S2, and insoluble-pellet from an equivalent number of cells were separated with SDS-PAGE and blotted with indicated antibodies.

To analyze the differences and similarities between the nucleosomes in the S1, S2, and pellet fractions in our protocol, we blotted for different histone PTMs in these fractions after normalization to the histone H3 levels in the samples (Figure 5A). For most PTMs, such as H3K4me3, H3K9me3, H3K27me3, K3K36me3, H3K79me2, and ub-H2A, their levels are quite comparable in the nucleosomes found in all three fractions. This suggests that the bulk of general chromatin is similarly distributed among the different fractions, and raises the confidence that the S1 + S2 fractions are representative of the bulk of genomic chromatin. However, we did observe that the majority of ubiquitylated H2B (detected by the mono-clonal H2Bub antibody) was found in the insoluble pellet fraction. We currently do not have an explanation for this observation but it is possible that H2Bub is enriched at chromatin domains or sub-nuclear compartments that are more resistant to MNase digestion. On the other hand, H2Bub is often associated with active chromatin in yeast and mammalian cells and; therefore, is not expected to be associated with repressive or compacted chromatin. When we examined the distribution of nuclear factors across the different fractions (see next section), we also find proteins associated with active transcription, such as Brd2 and RNA polymerase II (RNAPII) in the pellet fraction too, suggesting that the pellet still contains significant amounts of chromatin associated with active transcription. H2Bub may be associated with an active chromatin compartment that remains insoluble in this fractionation protocol, and further investigation will be needed to fully understand the molecular basis of this observation.

For additional characterization of the separated fractions, we also blotted for selected chromatin-interacting proteins including Brd2, USP39, USP7, PHF6, and RNAPII in these fractions. In this case, we did the comparisons using an equivalent number of cells instead of H3 normalization since different amounts of histones were found in these fractions. We found that chromatin-interacting proteins were mostly found in the S1 fraction as well as in the insoluble-pellet fraction (Figure 5B). Despite the fact that we are able to recover the majority of nucleosomes in the combined S1 and S2 fractions, much more of the chromatin-interacting proteins were found in the insoluble pellet compared to the S1/S2 fractions. Of interest, the heterochromatin marker, HP1α, and nucleolar marker, Fibrillarin, were only detected in the insoluble-pellet fraction by our assay, suggesting that heterochromatin and nucleoli are more resistant to MNase digestion (Figure 5B). As the S1 fraction contained more chromatin-interacting proteins compared to S2, our results suggest that one may opt to use S1 fraction only to assay chromatin-nuclear protein interactions, especially if the sensitivity of the detection of the nuclear proteins is an issue. However, we would recommend incubating the nuclei in S1 for a couple of hours after stopping the MNase reaction with EGTA (see step 9 below), as it helps to increase the yield of mononucleosomes in S1 versus S2 with no effect on the yield of mononucleosomes in the insoluble pellet (Figure 4C).

As a last note about this method, we have mostly expressed epitope-tagged histones in mammalian cells for purification of mono-nucleosomes because of the excellent immunoprecipitation efficiencies and capacities of the variety of antibodies against different epitope tags. Many studies have used histones that are tagged either at the C- or N-termini for detection and analysis, and expression of these histones, particularly in mammalian cells, generally does not affect their incorporation into chromatin, nor result in any deleterious effects. Although we have not validated the protocol for animal tissues, the technique should be adaptable for tissue samples. Once nuclei have been isolated from tissue samples using standard methods (Zaret, 2005; Krishnaswami et al., 2016), they can be treated with MNase for isolation of mononucleosomes using this protocol. However, it is important to note that, compared to tissue culture cells, the amount of tissues available for analyses will likely be limiting, which will impact on the final yield of nucleosomes. In addition, it should be possible to use various antibodies raised against endogenous histones or histone PTMs to immunoprecipitate endogenous nucleosomes akin to the native ChIP method. However, capturing sufficient nucleosomes for biochemical analyses will require antibodies that have excellent immunoprecipitation efficiencies and in larger scale compared to ChIP. The suitability of different histone antibodies for this nucleosome immunoprecipitation technique will have to be empirically determined in each case.

## Step-By-Step Protocol

### Isolation of Nuclei

1. Harvest 293T cells expressing FLAG-tagged histone of interest via trypsinization, resuspend in complete media and count cells (see Note 1 and 2).
2. Pellet cells via centrifugation (5 min, at 300 × *g*, 4°C), wash 1–2 times in cold 1X PBS and transfer cells in a 1.5 mL microcentrifuge tube. An aliquot can be taken out to prepare whole cell lysate to monitor the expression of FLAG-tagged histone.

Like any other protein analysis, samples and buffers should be kept on ice all the time unless otherwise stated.

3. Wash cells by thoroughly resuspending in 1 mL of Buffer A (for recipe see Table 1) containing protease inhibitors (see Note 3 and 4).

**TABLE 1:** Buffer A.

Stock	The amount for 40 mL	Final concentration
1M HEPES (pH = 8) (pH = 7.35 if using NEM)	400 μL	10 mM
1M KCl	400 μL	10 mM
1M MgCl_2_	60 μL	1.5 mM
1M Sucrose	13.6 mL	340 mM
50% Glycerol	8 mL	10%
1M DTT^1^	40 μL	1 mM
Autoclaved MilliQ H_2_O	Till 40 mL	

*^1^Add fresh.*
4. Pellet cells via centrifugation: 300 × *g*, 5 min, 4°C.
5. Remove and discard the supernatant. Thoroughly resuspend cells in 1 mL Buffer A as above and add Triton-X 100 to a final concentration of 0.2%. Mix and incubate on ice for 5 min.

Make 10% Triton-X 100 in sterile water and use 20 μL/1 mL of Buffer A to get a final concentration of 0.2%

6. Pellet nuclei: 600 × *g*, 5 min, 4°C. Keep and store the supernatant at −80°C, containing the cytoplasmic fraction, if desired.

### MNase Digestion

7. Wash the nuclei in 1 mL of Buffer A containing protease inhibitors (see Note 3 and 4).
8. Remove and discard the supernatant and resuspend the pellet in 500 μL of Cutting Buffer (for recipe see Table 2). Add MNase (Worthington) to the nuclei suspension at 1 U per 10^6^ cells and digest nuclei at 37°C for 30 min (see Note 5 and 6). Conditions for MNase digestion must be determined empirically for each cell line used (Note 6; Figure 6).

**TABLE 2:** Cutting buffer.

Stock	The amount for 40 mL	Final concentration
1M NaCl	600 μL	15 mM
1M KCl	2.4 mL	60 mM
1M Tris (pH = 7.5)	400 μL	10 mM
1M CaCl_2_	80 μL	2 mM
Autoclaved MilliQ H_2_O	Till 40 mL

**FIGURE 6:**
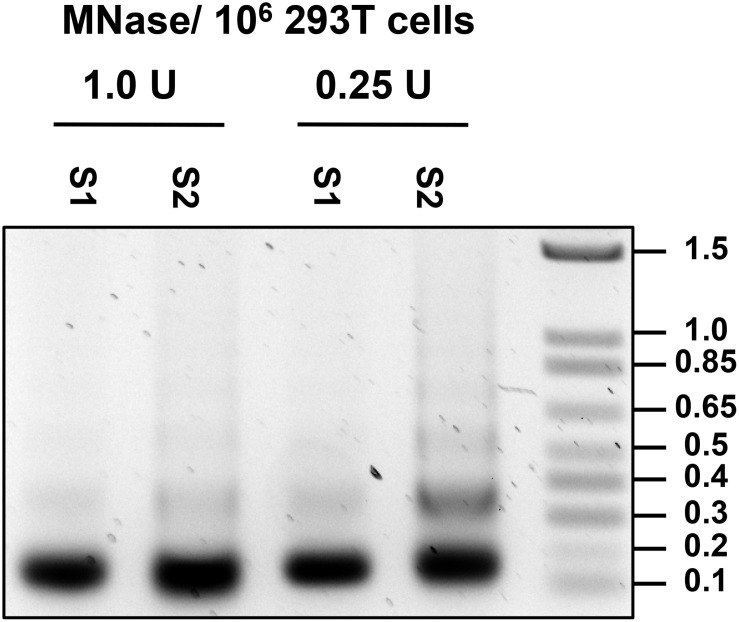
Optimization of MNase concentration. DNA was isolated from S1 and S2 fraction of nuclei treated with 1.0 and 0.25 U per 10^6^ cells, and 0.5 ug of DNA was run on 1.2% agarose gel.
9. Stop the MNase reaction by adding EGTA to a final concentration of 20 mM, mix and place on ice.

E.g., add 20 μL of 0.5 M EGTA stock/500 μL sample to get 20 mM final.

10. Pellet nuclei: 1300 × *g*, 5 min, 4°C. Transfer the supernatant to a fresh tube and store on ice (referred hereafter as the MNase-digested S1 fraction). A portion can be stored at −80°C for further analysis.
11. Resuspend the pellet with 500 μL of TE buffer (for recipe see Table 3) containing protease inhibitors (see Note 3 and 4). Incubate on ice for 30 min, mixing with a pipette every 10–15 min. Alternatively, samples can be rotated constantly using an end-over-end rotator at 4°C.

**TABLE 3:** TE buffer.

Stock	The amount for 40 mL	Final concentration
1M Tris (pH 8.0)	400 μL	10 mM
0.5M EDTA (pH 8.0)	80 μL	1 mM
Autoclaved MilliQ H_2_O	Till 40 mL
12. Spin samples 5 min at 13,000 × *g*, 4°C to pellet cell debris and insoluble material. Transfer supernatant to a new tube (referred here as the TE-soluble S2 fraction). A portion can be stored at −80°C for further analysis.
13. If desired, resuspend pellet in 250 μL of PBS and add an equal amount of hot 2X sample buffer (for recipe see Table 4), boil for 5 min to denature the proteins, and sonicate to resuspend proteins. Centrifuge at > 13,000 × *g* to remove insoluble material, if any. Transfer supernatant to a new tube (referred here as an insoluble-pellet fraction).

**TABLE 4:** 2X SDS sample buffer.

Stock	The amount for 40 mL	Final concentration
1M Tris (pH = 7.4)	0.8 mL	20 mM
0.5M EDTA (pH 8.0)	1.6 mL	20 mM
10% SDS	8 mL	2%
50% Glycerol	16 mL	20%
Autoclaved MilliQ H_2_O	Till 40 mL

### Immunoprecipitation (IP) of Nucleosomes

14. Transfer *exactly* 475 μL of S1 fraction from step 10 to a new tube. To re-adjust salt concentration, add 475 μL of 2X Buffer E *dropwise (1 drop every 2–3 s)* with constant mixing using the vortex at low speed (see Note 7 and 8; for recipe see Table 5).

**TABLE 5:** 2X buffer E.

Stock	The amount for 40 mL	Final concentration
1M HEPES (pH = 7.5)	1.2 mL	30 mM
4M NaCl	2.25 mL	225 mM
1M MgCl_2_	120 uL	3 mM
10% Triton X-100	1.6 mL	0.4%
50% Glycerol	16 mL	20%
Autoclaved MilliQ H_2_O	Till 40 mL
15. Transfer *exactly* 475 μL of S2 fraction from step 12 to a new tube. To re-adjust salt concentration of the S2 fraction, add 237 μL of 3X Buffer D *dropwise (1 drop every 2–3 s)* with constant mixing using the vortex at low speed (Note 7 and 8; for recipe see Table 6).

**TABLE 6:** 3X buffer D.

Stock	The amount for 40 mL	Final concentration
1M HEPES (pH = 7.5)	2.4 mL	60 mM
4M NaCl	4.5 mL	450 mM
1M MgCl_2_	180 uL	4.5 mM
0.5M EGTA (pH = 8)	48 uL	0.6 mM
10% Triton X-100	2.4 mL	0.6%
50% Glycerol	24 mL	30%
Autoclaved MilliQ H_2_O	Till 40 mL
16. Centrifuge tubes from Steps 14 and 15 (5 min at 13,000 × *g*, 4°C) and collect supernatant (salt-adjusted S1 and S2 fractions).
17. Pool the salt-adjusted S1 and S2 fractions in a 15 mL propylene tube to use as input for IP.
18. Transfer 50–100 μL from each pooled sample to a new tube and store at –80°C (save as input); use the remainder of the sample for IP.
19. Transfer 50% slurry of FLAG/M2 resin in a microcentrifuge tube using a wide bore tip. We typically use 20 μL per IP condition and wash a batch of beads enough for all IP conditions in an experiment (see Notes 9 and 10).

The current protocol uses a FLAG-tagged histone as an example. See Notes 11 and 12 for details on other antibodies and types of beaded support for purification.

20. Pellet the beads by centrifuge (700 × *g* for 15 s at 4°C) and discard the supernatant.
21. Wash the beads twice in 1X buffer D by pelleting (700 × *g* for 15 s at 4°C) and gently resuspending with the buffer without pipetting up and down (see Note 13).
22. After the final wash, resuspend the beads with 1X buffer D to make 50% slurry.
23. Incubate 20 μL of 50% slurry with the IP sample from step 17 overnight at 4°C in 15-mL propylene tubes. Keep samples rotating constantly using an end-over-end rotator.
24. After overnight incubation, pellet the beads by centrifugation (700 × *g* for 15 s at 4°C). Save supernatant, if desired (= UNBOUND).
25. Resuspend beads in 500 μL 1X buffer D and transfer the beads to a microcentrifuge tube using a wide-bore 1000 μL tip for subsequent washing steps.
26. Repeat the step above using 500 μL 1X buffer D to transfer leftover beads from 15 mL tube to the same microcentrifuge.
27. Wash pelleted down beads 2–4 times in 1X Buffer D by pelleting (700 × *g* for 15 s at 4°C) and gently resuspending with the buffer without pipetting up and down (see Note 13).
28. Wash the beads another 2–4 times in 1X Buffer D + 0.5% Triton X-100 without pipetting up and down (see Note 13).
29. The immunoprecipitated nucleosomes and proteins can be processed for mass spectrometry or immunoblot analysis at this point.

### SDS-PAGE and Immunoblot Analysis

30. Gently resuspend the beads collected from step 28 in 20 μL 2X SDS sample buffer and boil for 5 min to denature the protein followed by centrifugation to pellet the beads: 700 × *g* for 15 s at room temperature (see Note 14). Also, boil the input from step 18 in an equal volume of 2X SDS sample buffer.
31. Run input and IPs on 15% SDS-PAGE and stain with Coomassie stain for 1 h followed by destining overnight to confirm the presence of mononucleosomes.
32. Normalize the IP’d nucleosomes from different samples by the total histones IP’d (e.g., based on Coomassie-stained histone bands) or by H3 (by immunoblot analysis). Once the amount of H3 IP’d across different samples has been normalized, perform immunoblot analysis using antibodies against Flag, different histone PTMs, or other proteins of interest to ascertain the relative amounts of proteins/histone PTMs that are associated with or co-IP with the immunoprecipitated nucleosomes.

### Extraction of DNA From MNase-Digested S1 and TE-Soluble S2 Fractions

1. Aliquot 100 μL each of S1 and S2 fractions in 1.5 mL microtubes. Add 1 μL of 10 mg/ml Proteinase K and incubate at 37°C for 3 h to overnight.
2. Add an equal volume of Phenol: Chloroform/Isoamyl alcohol (24:1), mix well by rocking and centrifuge at high speed for 5 min.
3. Collect the top aqueous phase of each tube and transfer to a new 1.5 mL microtube with an equal volume of Chloroform/Isoamyl alcohol 24:1. Vortex and centrifuge at high speed for 5 min.
4. Collect the top aqueous phase of each tube and transfer to a new 1.5 mL microtube. Centrifuge at high speed for 5 min to separate aqueous phase from carried over organic phase, if any.
5. Collect the top aqueous phase in a new 1.5 mL microtube and precipitate DNA by adding 1/10 volume of 3M Sodium acetate pH 5.2 and twice the volume of ice-cold ethanol.
6. Incubate at −20°C for 2 h or overnight and centrifuge at high speed at 10 min to pellet the DNA.
7. Discard the supernatant and wash the pellet with 500 μL of 70% ethanol. Centrifuge at high speed for 5 min.
8. Discard the supernatant and air-dry the pellet for 15 min.
9. Resuspend the pellet in 100 μL of TE buffer. If necessary, allow the DNA to dissolve at 4°C for a couple of hours and quantify DNA using nanodrop.
10. Run 0.5 ug of DNA per condition in 1.2% Agarose gel to access the level of shearing of chromatin.

### Notes

1. Cell culture: Avoid cells that have become over-confluent. Passage cells while still sub-confluent. We typically passage 293T cells twice a week.
2. The required number of cells depends on the target application. We typically IP from a confluent 15 cm plate of 293T cells (roughly 40–50 million cells) and 1/20 of eluate is used for Coomassie gel and blotting PTMs while 1/4 of eluate is used for blotting nucleosome-interacting proteins.
3. To avoid protein degradation, add the following protease inhibitors to all the buffers at a stated concentration just prior to use; PMSF 200 uM, Aprotinin 1 ug/ml, Leupeptin 10 uM, Pepstatin 1 uM.
4. To prevent loss of PTMs, add the following additives to all the buffers, depending on the experiment; an inhibitor of deubiquitinases *N*-ethylmaleimide (NEM) at 10 mM final concentration, HDAC inhibitor Sodium Butyrate at 5 mM final concentration, or phosphatase inhibitor Microcystin-LR at 0.1 uM final concentration.
5. MNase (from Worthington) is resuspended in 5 mM Tris-HCl (pH 7.5)/10 uM CaCl_2_ at 50 U/μL, aliquoted, and stored at −20°C. Freeze-thaw cycles should be kept to a minimum. As different manufacturers define units of MNase differently, special attention should be paid to the unit definition and conversion formula if using a different source.
6. Optimal MNase amount needed for chromatin digestion must be determined empirically for each cell line used, source of MNase and specific batch of MNase. This is done by treating cells with varying amounts of MNase and DNA extracted from the S1 and S2 fractions for each condition are run on a 1.2% Agarose gel (see section “Extraction of DNA From MNase-Digested S1 and TE-Soluble S2 Fraction”). Mononucleosome preparation typically should consist of mononucleosomes with a small amount of di-nucleosomes still present (Figure 6). Special attention should be paid to avoid over-shearing as MNase is able to cleave DNA internally at ∼10 bp interval where it is exposed to the outer surface of the core nucleosome (Clark, 2010).
7. If 3X Buffer D and 2X Buffer E are prepared ahead of time, store at 4°C; incubation on ice may cause salts to precipitate.
8. Vortexing the samples in a microcentrifuge tube with the lid open can lead to a sample spill. So the optimal vortex speed can be first determined using a “dummy” sample.
9. Mix the beads well by rotating the original container. For ease of handling and to avoid mechanical damage, pipette the beads with wide bore tips that are available commercially or can simply be generated by cutting off the end of the regular pipette tip.
10. We typically use 20 μL of beads that may be increased depending on the expression level of the target protein. Using < 20 μL beads can present visualization difficulties. The pellet of beads can be better visualized against a light lamp or dark background.
11. For IP of biotinylated proteins, use Streptavidin-Agarose (Sigma, cat. no. S1638) or Streptavidin Sepharose High Performance (GE, cat. no. 17-5113-01). For IP of Strep-tag tagged Histones, Strep-Tactin XT Superflow (IBA, cat. no. 2-4010) can be used. Alternatively, Sepharose or Magnetic Protein G/A beads along with specific antibodies against a histone protein or PTM may be used (see Note 12 for further details). The choice of Protein G or A beads depends on the affinity of the beaded support with the isotype of the antibody being used. For further information refer to Zhang and Chen (2001) and Bonifacino et al. (2016).
12. If histone- or histone PTM-specific antibodies are used, particular attention should be paid to the specificity of each antibody used and they should all be carefully validated (Bordeaux et al., 2010; Laflamme et al., 2019). Histone PTM antibodies are generally raised using modified peptides but the presence of other PTMs on endogenous histones may interfere with the detection by the antibody (Egelhofer et al., 2011; Rothbart et al., 2015). Consultation with histone antibody databases such as http://www.histoneantibodies.com/ or https://www.encodeproject.org/antibodies/ can offer insights into possible occlusions of the targeted epitopes by surrounding modifications for tested commercial antibodies. Another issue to be considered is that antibodies need to be checked for cross-reactivity with other proteins or histones that have identical or similar motifs (for example, the ARKS amino acid motif flanking K9 is identical to the sequence around K27 of histone H3). Testing the loss of reactivity of histone PTM antibodies toward histones where the specific sites of modification are mutated could be useful for confirming their specificities. Lastly, as this protocol is similar to native-ChIP, antibodies suitable for native ChIP/ChIP-seq may be a good starting choice for selecting antibodies.
13. Avoid losing any beads during washing step by careful aspiration and by not disturbing the pelleted beads. The supernatant can be aspirated with 200 μL micropipette tips attached to a vacuum line of an aspirator. The tip should be advanced along the side of the tube until it reaches just above the beads. After the last wash, pipette the last few μL of wash buffer using a narrow-bore flat tip, if available.
14. Mix the beads with elution buffer by gently tapping the tube and not by pipetting up and down to avoid losing the beads. It is unnecessary to remove the eluate from the beads and can be stored along with beads at −20°C.

## Typical Results

**FIGURE 7:**
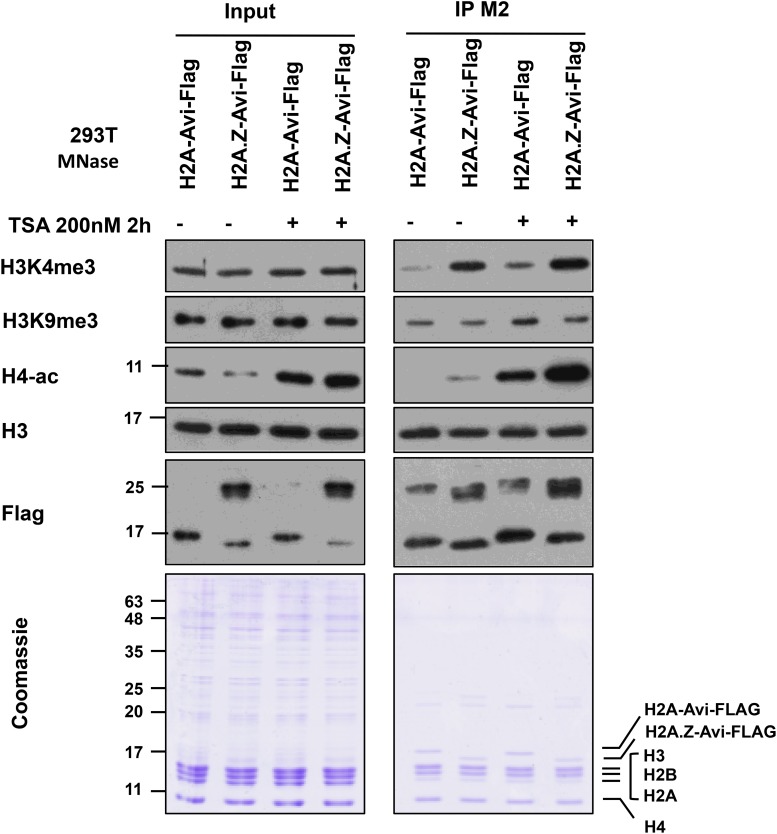
Immunoprecipitation of H2A and H2A.Z containing mononucleosomes for analysis of H3K4me3, H3K9me3, and H4ac. 293T cells (7 × 10^6^) were plated in a 15 cm plate. Next day, the media were changed and cells were transfected with (pcDNA3) H2A-Avi-Flag and H2A.Z-1-Avi-Flag. 46 h later, cells were treated with ethanol or TSA at 200 nM for 2 h before harvesting. Mononucleosomes were prepared 48 h post-transfection and immunoprecipitated using the current protocol. For Coomassie and blots, 1/350 of Input and 1/20 of the eluate was run on 10–15% SDS-PAGE and blotted with indicated antibodies.

An example of mononucleosome IP analysis for H3 PTMs co-existing with histone variant H2A.Z is shown in Figure 7. We expressed H2A-Avi-Flag or H2A.Z-Avi-Flag in transfected 293T cells followed by mononucleosome preparation. H2A and H2A.Z containing mononucleosomes were immunoprecipitated with FLAG-M2 resin using the current protocol. The eluate was normalized by the amount of H3 and blotted for different histone PTMs. As seen before (Draker et al., 2012), we observed that H3K4me3 and H4-ac were enriched on H2A.Z containing mononucleosomes while H3K9me3 was enriched on H2A containing mononucleosomes. This difference was also observed in hyperacetylated histone condition following treatment of cells with the histone deacetylase inhibitor trichostatin A (TSA). As TSA results in the overall acetylation of histones, we observed an increase in acetylation of histone H4 following TSA treatment (Figure 7).

## Time Considerations

Once the cells expressing FLAG-tagged histone are ready, isolation of nuclei takes 1 h followed by the preparation of mononucleosomes in 1.5 h. Generally, we perform the IP the same day, which can be incubated for 1 h or until the next day (i.e., overnight). Washing of beads and elution may take 1 h the next day. So mononucleosome IP is typically completed over 2 days. The eluate may be analyzed immediately (e.g., resolved by SDS-PAGE) or stored at −20°C.

## Discussion

We have used this mononucleosome IP protocol to isolate the histone variant H2A.Z-containing nucleosomes from human cells and performed proteomic screen with these nucleosomes to identify proteins that co-purify with H2A.Z-1 nucleosomes (Draker et al., 2012). Some of our validated hits (e.g., Brd2, PWWP2A, PHF14) were also identified in other independent mononucleosome IP screens (Kim et al., 2013; Vardabasso et al., 2015; Surface et al., 2016; Punzeler et al., 2017). However, we note that there are variations in the interactors identified by these different studies, possibly due to the differences in the protocol and/or cell line used. For that reason, we characterized the relative abundance of selected histone PTMs and chromatin-binding proteins and presented the data as well as a detailed protocol in this article. The use of mononucleosome IP has been gaining popularity and has been used to identify proteins interacting with other histone variant-containing nucleosomes such as macroH2A and H2A.Bbd (Sansoni et al., 2014; Sun et al., 2018) as well as CENP-A/cenH3 and H3.Y (Fang et al., 2015; Zink et al., 2017). Apart from histone variants, this approach was also used to demonstrate the YEATS domain as a crotonyllysine reader by showing a preference of AF9 YEATS domain for crotonylated over acetylated lysines in histone H3 (Li et al., 2016). In addition, we and others have used this method to examine the relative abundance of histone PTMs on core or variant histones in the purified mononucleosomes. This approach allows the identification of PTMs on different histones that are co-enriched within the nucleosome context as well as possible cross-talks amongst different PTM combinations. For example, it has been demonstrated that H2A.Z and H3.3 containing mononucleosomes are enriched for activating histone PTMs such as K4 methylated H3 (Viens et al., 2006; Sarcinella et al., 2007; Won et al., 2015), whereas macroH2A containing mononucleosomes are enriched for repressive histone PTMs like K9 methylated H3 (Won et al., 2015). In addition, this method has also been used to dissect the cross-talk between macroH2A1 and H2B acetylation (Chen et al., 2014; Ruiz and Gamble, 2018), and H2A.Z ubiquitylation with H3K27me3 (Ku et al., 2012; Surface et al., 2016; Ng et al., 2019). Lastly, the incorporation of specific core or variant histones into chromatin has been demonstrated by the nucleosome-immunoprecipitation method (Kanda et al., 1998; Wiedemann et al., 2010; Lau et al., 2011; Ruiz and Gamble, 2018). Altogether, these studies have shown a general utility of this method.

One other area in which mononucleosome immunoprecipitation has been particularly useful is the study of oncohistones i.e., mutations at K27, and K36 in histone H3 and its variants that have been linked to oncogenesis. In fact, expression of H3.3 K27M, and K36M mutants results in global loss of methylation status of endogenous H3 at the corresponding lysine (Mohammad and Helin, 2017). Additional studies used mononucleosome immunoprecipitation to demonstrate that H3.3K27M mutant containing mononucleosomes have reduced H3K27me3 and increased H3K27ac on the wild type endogenous H3 partner, and are enriched for the H3K27 methyltransferase EZH2 (Bender et al., 2013; Chan et al., 2013; Lewis et al., 2013; Fang et al., 2017). Indeed, loss of H3 methylation results in the formation of H3K27M-K27ac heterotypic nucleosomes that are enriched with acetyl-binding bromodomain-containing protein 1 (BRD1) and BRD4 proteins (Herz et al., 2014; Piunti et al., 2017). Similarly, H3.3K9M mutant containing mononucleosomes were observed to have decreased K9 methylation at endogenous H3, and increased association with the H3K9 demethylase KDM3B and the H3K9/K56 deacetylase SIRT6 (Herz et al., 2014). Subsequently, it was also demonstrated that H3.3K36M mutant containing mononucleosomes displayed reduced H3K36me2/3 on endogenous H3 and are enriched for H3K36 methyltransferases SetD2, Nsd1, Nsd2, and MMSET (Fang et al., 2016; Lu et al., 2016).

An advantage of this protocol is that it can potentially identify proteins that interact with nucleosomes through multiple PTMs on different histones of the same nucleosome. These proteins may not otherwise be identified using the standard co-IP procedure. Brd2 has been shown to interact with H2A.Z-1 and H2A.Z-2 nucleosomes through a combinatorial interaction with H2A.Z and acetylated H4 (Draker et al., 2012; Vardabasso et al., 2015). Conversely, ubiquitylation of H2A.Z was found to antagonize Brd2 binding to H2A.Z nucleosomes (Surface et al., 2016). Such an approach has also been used to confirm the multivalent interaction of PWWP2A with H2A.Z nucleosomes (Punzeler et al., 2017). Lastly, IP of mononucleosomes was used to demonstrate the existence of asymmetrically modified nucleosomes in addition to symmetrically modified nucleosomes, with respect to H3K27me2/3 and H4K20me1 (Voigt et al., 2012).

As with any experimental approaches, there are limitations and drawbacks to this mononucleosome IP method. First, we rely on the expression of tagged histones (e.g., Flag-tagged H2A.Z) in transfected cells in order to use the highly efficient Flag M2 beads to capture the tagged histone-containing nucleosomes. Immunoprecipitation of endogenous histones is always desirable, and in theory this protocol should also work with antibodies against specific histones or histone PTMs. However, as mentioned previously, it depends on the immunoprecipitation efficiencies of the antibodies against the intended targets, which will have to be individually tested and optimized. For our purposes, we chose to express tagged histones because of the advantage of well-characterized and highly efficient antibodies against epitope tags. In our hands, the expression levels of the tagged H2A.Z is fairly comparable to the endogenous H2A.Z protein levels (data not shown), and we have not observed any interference of histone incorporation, nor growth defects or deleterious effects in these tagged H2A.Z-expressing cells. We also typically choose to place the epitope tag on the C-terminus of histones, but it is possible that, in some cases, the presence of a tag may interfere with normal PTM profile and thus the association of histone binding proteins. If necessary, one could test for potential interference effects by comparing results using N-terminus vs. C-terminus tagged histones. All these parameters should be carefully monitored for different histones, histone variants, and tags. Second, as noted earlier, it would be highly desirable to immunoprecipitate nucleosomes using histone-PTM-specific antibodies; however, these are not always available (see note 12 for considerations for choosing the right antibody for the assay). To overcome this limitation, we have previously developed a technique named BICON (biotinylation-assisted isolation of co-modified nucleosomes) whereby we co-modify targeted histone substrates with a fusion of a histone-modifying enzyme and the *E. coli* biotin ligase BirA, and then use streptavidin-couple reagents to pull down the biotinylated and enzyme-modified histone/nucleosome [see (Lau and Cheung, 2013) for specific details]. As proof of principle, we co-expressed H3.3 with a 15 amino acid tag called Avi-tag that is the specific biotinylation target of BirA, along with an MSK1-BirA fusion (MSK1 is a well-characterized H3 kinase) in human cells, and demonstrated efficient purification of nucleosomes that contain H3.3 that are both biotinylated and phosphorylated.

In this protocol, we rely on micrococcal nuclease digestion to release mono-nucleosomes from bulk chromatin. We have chosen an MNase concentration that generates mostly mono-nucleosomes without excessive digestion, but we have not tested whether higher amounts of MNase could release more nucleosomes and chromatin-binding proteins from the pellet fraction. Indeed, chromatin is sensitive to MNase at varying degrees. Although the precise reasons for differential sensitivity of individual nucleosome to MNase are unknown, preference of MNase toward adenine/thymine (A/T) nucleotides may be a contributing factor. It has been reported that over-digestion can result in artificial depletion of A/T rich genomic regions (Kensche et al., 2016). As shown in Figure 5 of this article, there are significant amounts of chromatin-bound proteins, as well as some nucleosomes, that remain in the insoluble pellet after MNase digestion and hence are not used for immunoprecipitation. Moreover, some proteins (e.g., HP1α, Fibrillarin) and modified histones (e.g., H2Bub) appear to be almost exclusively found in the insoluble pellet. Therefore, this mono-nucleosome immunoprecipitation approach will not be suitable for examining nuclear factors or histones that are exclusively retained in the insoluble pellet. The precise explanation of these observations is not known, but there could be MNase-resistant or insoluble fractions of chromatin that are enriched with specific types of chromatin. Along that line of thinking, a previous study discovered and characterized a sonication-resistant fraction of chromatin in their ChIP-seq method that is enriched for proteins/genomic sequences associated with unique subtypes of heterochromatin, and is refractory to cell reprogramming (Becker et al., 2017). Therefore, similar genomic/proteomic analyses of the pellet fraction from our fractionation protocol may reveal new insights into subtypes of chromatin domains and compartments as well.

## Materials

### Reagents

1 kb Plus DNA ladder (Thermo Fisher Scientific, cat. no. 10787-018)
293T cells cultured on 145-mm plates (expressing FLAG-tagged Histone protein)
6X Gel Loading Dye, Purple (NEB, cat no. B7024)
Agarose (Fisher BioReagents, cat. no. BP160)
Anti-Flag M2 Affinity Gel (Sigma-Aldrich, cat. no. A2220)
Aprotinin (Sigma-Aldrich, cat. no. A1153)
CaCl_2_ solution (Sigma-Aldrich, cat. no. 442909)
Chloroform (Caledon, cat. no. 3001-2)
Dithiothreitol (DTT; Fisher BioReagents, cat. no. BP172)
DMEM Media, high glucose (HyClone, cat. no. SH30243FS)
EDTA (Sigma-Aldrich, cat. no. EDS)
EGTA (Sigma-Aldrich, cat. no. E4378)
Equilibrated Phenol solution (Sigma-Aldrich, cat. no. P4557)
Ethanol (Commerical Alcohols, cat. no. P006EAAN)
Fetal Bovine Serum (Sigma-Aldrich, cat. no. F1051)
Glycerol (Fisher BioReagents, cat. no. BP229)
HEPES (Fisher BioReagents, cat. no. BP310)
Isoamyl alcohol (Fisher BioReagents, cat. no. BP1150)
KCl (Sigma-Aldrich, cat. no. P9541)
Leupeptin (Sigma-Aldrich, cat. no. L2884)
MgCl_2_ (Sigma-Aldrich, cat. no. M9272)
Micrococcal Nuclease (Worthington Biochem, cat. no. LS004798)
Microcystin-LR (Cayman, cat. no. 10007188)
NaCl (Fisher BioReagents, cat. no. BP358)
*N*-ethylmaleimide (NEM; Sigma-Aldrich, cat. no. E3876)
Pepstatin (Sigma-Aldrich, cat. no. P5318)
Phenylmethylsulfonyl fluoride (PMSF; Sigma-Aldrich, cat. no. P7626)
Phosphate buffered saline (PBS; Wisent, cat. no. 311-010-CL)
Phenol:chloroform:isoamyl alcohol (UltraPuro, Thermo Fisher Scientific, cat. no. 15593-031)
Chloroform/Isoamyl alcohol 24:1 (CI)
Proteinase K (Sigma-Aldrich, cat. no. P2308)
RedSafe Nucleic Acid Staining Solution (iNtRON Biotechnology, cat. no. 21141)
Sodium acetate (Sigma-Aldrich, cat. no. S2889)
Sodium Butyrate (Sigma-Aldrich, cat. no. B5887)
Sucrose (Bioshop, cat. no. SUC700)
Tris (Fisher BioReagents, cat. no. BP152)
Triton X100 (Sigma-Aldrich, cat. no. T9284)
Trypsin 0.05%, EDTA 0.53MM in HBSS, 1X (Wisent, cat. no. 325-542)

### Antibodies

Anti-FLAG-M2 affinity gel (Sigma-Aldrich, cat. no. A2220)
Anti-PHF6 (Bethyl, cat. no. A301-450A)
Anti-USP39 (Abcam, cat. no. ab131332)
Anti-USP7 (Bethyl, cat. no. A300-033A)
Anti-Brd2 (Abcam, cat. no. ab3718)
Anti-RNA PolII (Covance, cat. no. MMS-126-R)
Anti-HP1α (Santa-Cruz, cat. no. sc-47701)
Anti-Fibrillarin (Abcam, cat. no. ab5821)
Anti-Tubulin (Sigma-Aldrich, cat. no. T6074)
Anti-H3 (Abcam, cat. no. ab1791)
Anti-H2A.Z (Active Motif, cat. no. 19113)
Anti-H3K4me3 (Millipore, cat. no. 07-030)
Anti-H3K9me3 (Millipore, cat. no. 07-442)
Anti-H3K27me3 (Millipore, cat. no. 07-449)
Anti-H3K36me3 (Abcam, cat. no. ab9050)
Anti-H3K79me2 (Millipore, cat. no. ABE459)
Anti-ub-H2A K119 (Cell Signaling, cat. no. 8240)
Anti-ub-H2B (Medi Mabs, cat. no. MM-0029)
Anti-H4-ac (Millipore, cat. no. 06-946)

### Equipment

Vacuum aspirator
Ice and ice bucket
End-to-end rotator
1.5-ml microcentrifuge tubes
15-ml and 50-ml propylene tubes
Agarose gel apparatus
Electrophoresis power supply
Micropipettes (e.g., Pipetman, Gilson)
Refrigerated microcentrifuge (e.g., Eppendorf 5415R)
SDS-PAGE running and transfer apparatus
Tissue culture dish, 145 mm (Grenier Bio-One, cat. no. 639160)
UV Spectrophotometer (e.g., NanoDrop 2000, Thermo Scientific)
Water bath set at 37°C.

## Data Availability Statement

All datasets generated for this study are included in the article/supplementary material.

## Author Contributions

PC conceived the original idea and supervised the project. MN performed initial optimization of the protocol and KK performed the experimental work presented in this manuscript. KK wrote the first draft followed by revision with contributions from MN and PC.

## Conflict of Interest

The authors declare that the research was conducted in the absence of any commercial or financial relationships that could be construed as a potential conflict of interest.
